# Experimental Study on Shear Performance of Longitudinal Joints in Prefabricated Invert Arch for Mountain Mining Method Tunnels

**DOI:** 10.3390/ma18133025

**Published:** 2025-06-26

**Authors:** Shiqian Zhang, Minglei Ma, Chang Li, Peihuan Ye, Zongping Chen

**Affiliations:** 1 China Construction Eighth Engineering Division Co., Ltd., Shanghai 200112, China; mingleimacivil@163.com (M.M.); lzb1006@126.com (C.L.); 2 School of Civil Engineering and Architecture, Guangxi University of Science and Technology, Liuzhou 545006, China; peihuan.ye@gxust.edu.cn; 3 College of Civil Engineering and Architecture, Guangxi University, Nanning 530004, China; zpchen@gxu.edu.cn; 4 Key Laboratory of Disaster Prevention and Structure Safety of the Ministry of Education, Guangxi University, Nanning 530004, China

**Keywords:** tunnel assembly inverted arch, longitudinal joint, shearing performance, bolted connection, shear-bearing capacity calculation method

## Abstract

In order to improve the efficiency of highway tunnel construction and ensure the construction quality, the design concept of a prefabricated inverted arch and partial cast-in-place lining of highway tunnels by a mining method is put forward. During the assembly of prefabricated tunnel invert arches, the longitudinal joints between adjacent invert sections were subjected to shear forces due to the combined effects of the invert’s self-weight and construction equipment loads. This study investigated the shear performance of these longitudinal joints under construction loads, with a particular focus on the influence of bolt-tightening torque. Three longitudinal joint specimens were designed and fabricated, varying the bolt-tightening torque as a key parameter, and subjected to shear tests. The failure modes, load–slip behavior, and shear capacity of the joints were analyzed in relation to the tightening torque of high-strength bolts. The results indicate that when the bolt-tightening torque was set to 50% and 70% of the standard torque, the upper bolts of the joint sheared off, while the threads of the lower bolts were damaged. When the torque reached the standard value, all bolts were sheared off. The ultimate shear capacity of the longitudinal joints increased with higher bolt-tightening torque, with the optimal torque range identified as 70% to 85% of the specified standard. Ultimately, a method of calculation for evaluating the shear-bearing capacity of inverted arch longitudinal joints was proposed, with computational outcomes demonstrating a conservative bias that aligns with structural safety requirements.

## 1. Introduction

Tunnel traffic engineering serves as a critical component of modern infrastructure, significantly enhancing regional connectivity, transportation efficiency, and cross-border infrastructure integration. Among contemporary construction methods, prefabricated building technology has emerged as a superior alternative to traditional cast-in-place techniques, offering notable advantages in efficiency, sustainability, and adaptability. Specifically, prefabrication demonstrates greater resilience to challenging construction environments, including extreme weather and complex geological conditions, while simultaneously reducing costs and accelerating project timelines [1,2]. At present, the research and application of prefabricated building technology are more common in urban subway tunnel projects. The fully prefabricated Changchun subway built in 2012 controlled the assembly error within 2 mm, which greatly improved the overall stability and safety of the structure [3]. In 2019, the prefabricated cast-in-place composite arch shell technology was first applied to the superimposed prefabricated subway station built on Wuzhong Road in Shanghai, which effectively solved the technical problems of the limited construction site and difficult transportation of large, prefabricated components in this area [4]. These practical cases have achieved good results. Lin et al. [5] studied the mechanical properties of the tenon joint at the vault and its spring of the assembled station structure, and the research showed that increasing axial force significantly enhanced both the load-bearing capacity and deformation resistance of these critical structural components. Jiang et al. [6] proposed a research theory of deformation characteristics considering the difference of bending stiffness based on experiments. Tao et al. [7,8] conducted a shaking table test of a fabricated subway station, and the research showed that the fabricated structure was less damaged than the cast-in-place structure, showing good seismic performance. However, there are relatively few studies and applications in highway and railway tunnel construction. As an important direction for the development of building structures in the future, prefabricated structures are an important way to realize the industrialization of underground engineering. Therefore, it is necessary to continue to develop the prefabricated assembly technology of highway and railway tunnels.

The key component of the precast concrete structure is its connection technology. The connection of the prefabricated tunnel lining structure can be divided into the circumferential joint and longitudinal joint. The connection between the annular segments of the same ring is called the circumferential joint, and the joint between the segment rings is the longitudinal joint. The common joint forms of prefabricated underground structures include the bolt joint structure [9,10,11,12], mortise joint structure [13], and steel sleeve grouting joint structure [14]. As a widely used joint structure, the bolt joint structure often has problems, such as interface strength weakening and water leakage in the connection between components [15,16], which in turn affects the stability and safety of the structure. Therefore, it is very important to study its mechanical performance. Through the study of bolted joints in shield tunnels, it was found that the stress distribution of bolt shear deformation was mainly concentrated on the thread edge and the outer surface of the joint [17]. Huang et al. [18] discussed the mechanical performance index changes of the joints of the prefabricated frame tunnel lining with or without bolts. The results showed that the ultimate bearing capacity of the reinforced bolts on the joint surface was 13.44% higher than that without bolts, and the crack opening and compression were reduced by dozens of times. Adding bolts can effectively improve the mechanical properties of tunnel lining joints. Han et al. [19,20] proposed a numerical analysis model for calculating the shear stiffness of bolts, which had a positive impact on obtaining the stress and deformation state of circular segments. Furthermore, considering the influence of relative displacement caused by the gap between bolts and bolt holes, a calculation model of the shear strength of circular joints in the tunnel segment lining was proposed. The longitudinal joint is the key factor to determine the longitudinal mechanical properties of the tunnel structure. It is very important to study the mechanical properties of the longitudinal joint connection for structural operation safety. Klappers et al. [21] proposed a beam-spring coupling and shell element analysis model considering the longitudinal change in structural stiffness, which can better reflect the stress change of the longitudinal joint but still cannot accurately express its spring stiffness. Wang et al. [22] proposed the improved four-stage analytical model of the shield tunnel lining, which can accurately obtain the joint bending stiffness. Geng et al. [23] carried out a local full-scale test on the longitudinal joints of shield tunnels and found that when the longitudinal tension was the largest, the segment structure was destroyed before the bolt, and the bolt did not reach the yield strength. The change in the longitudinal joint opening was more sensitive to the axial force. Zhang et al. [24] studied the mechanical behavior of longitudinal joints of shield tunnels under large deformation conditions, and found that concrete cracking, spalling, or spalling and fracture failure of bolts and threads occurred on the compression side of the joints.

The mining method is a traditional tunnel construction method with drilling and blasting excavation as the core, and the assembled inverted arch combined with the mining method can solve the shortcomings of the traditional mining method, such as slow construction speed and inverted arch ring lag. To ensure the longitudinal performance of prefabricated tunnels, priority must be given to evaluating the adaptability and safety of precast components under construction loads during the assembly process. Building on the aforementioned research, this study focuses on a highway tunnel project in southern China. A 1/2 scale prefabricated invert segment is designed based on the original tunnel height, and a series of quasi-static loading tests are conducted to investigate the mechanical behavior of longitudinal bolted connections during the construction process. Based on the experimental findings, a calculation method for the bearing capacity of the connection is proposed, with the aim of providing a reference for similar engineering applications and further scientific research.

## 2. Experimental Program

### 2.1. Experimental Background

**Figure 1:**
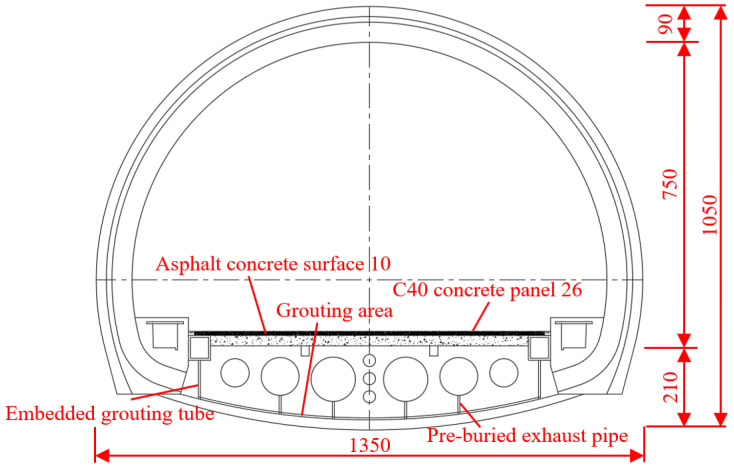
Horseshoe-shaped lining cross-section (unit: cm).

**Figure 2:**
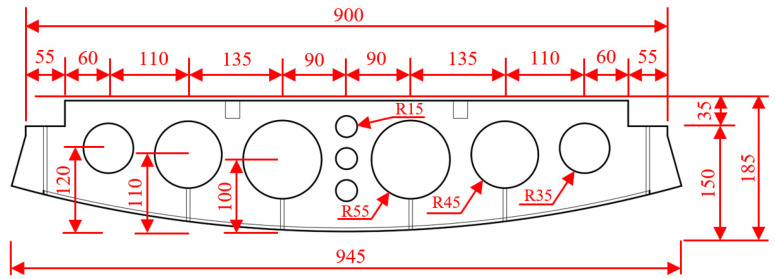
Dimensional detailing of precast invert arch (unit: cm).

This article is based on a mountain tunnel project in the south of China, where the tunnel form is horseshoe-shaped, as shown in Figure 1. The thickness of the secondary cast-in-place lining of the arch wall is 0.045 m. The inverted arch of the tunnel adopts a prefabricated concrete inverted arch block. The length of a single inverted arch block along the longitudinal direction of the tunnel is 9.45 m, the width of the block is 1.00 m, and the height is 1.85 m. When the inverted arch block is prefabricated, 10.00 m^3^ concrete is poured at one time, the concrete strength grade is C40, and the weight of the inverted arch block is 25.00 t. The detailed size is shown in Figure 2.

### 2.2. Construction Stress Characteristics of Longitudinal Joint

**Figure 3:**
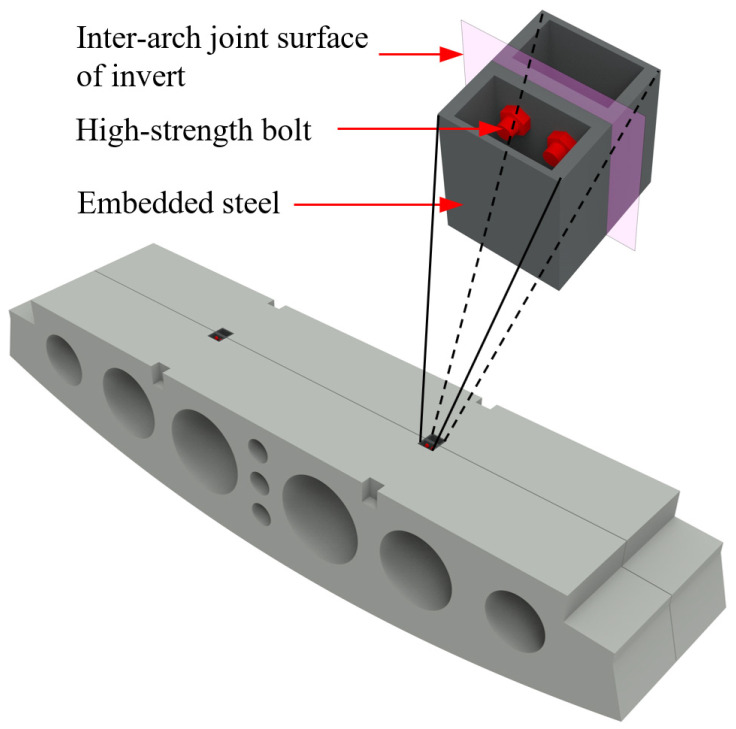
Longitudinal joint configuration in invert arch.

**Figure 4:**
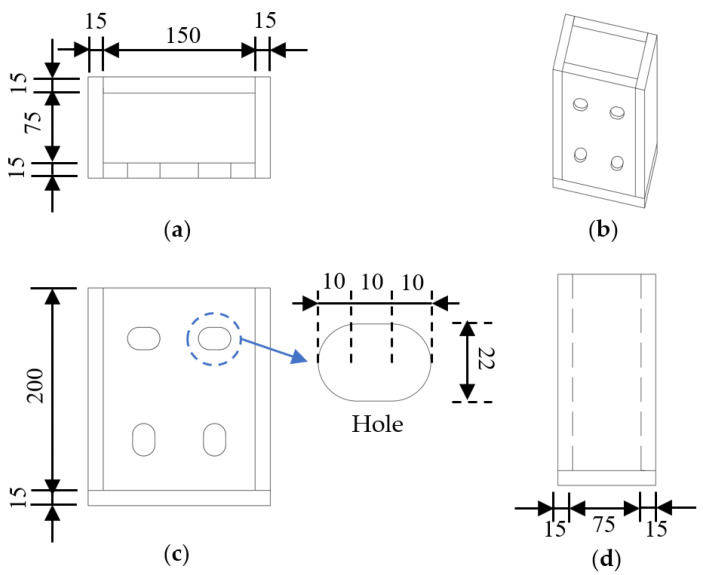
Three views and the three-dimensional structure of the embedded steel box (unit: mm): (**a**) vertical view, (**b**) elevation drawing, (**c**) front view, and (**d**) side view.

The experimental study relied on the actual tunnel project, employing horizontally bolted joints to connect adjacent prefabricated invert segments. Each joint assembly consisted of four high-strength bolts and two embedded steel connection boxes, as shown in Figure 3. The steel boxes were positioned at the crown of each invert segment and welded to the internal reinforcement framework, and the three views and the three-dimensional structure are shown in Figure 4. During tunnel assembly, adjacent invert segments were securely joined by torque-controlled tightening of the high-strength bolts.

**Figure 5:**
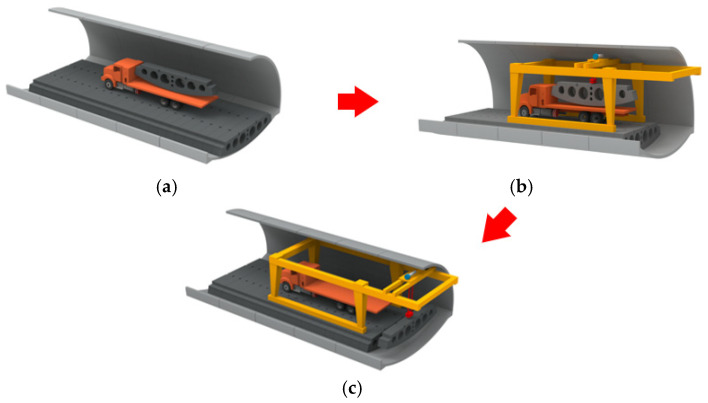
Dimensional details of the precast invert arch: (**a**) transportation, (**b**) lifting, and (**c**) assembly.

The construction scheme of the assembled inverted arch structure in practical engineering is implemented in [25]. The main processes of inverted arch assembly are transportation, lifting, and assembly, which are detailed in Figure 5. It can be seen from Figure 4 that the prefabricated inverted arch members provide driving conditions for various construction equipment during their assembly process when the secondary lining of the tunnel arch wall has not yet been constructed. Under these conditions, the longitudinal joints may be subjected to shear force or the combined action of shear force and bending moment.

### 2.3. Specimen Design and Fabrication

**Figure 6:**
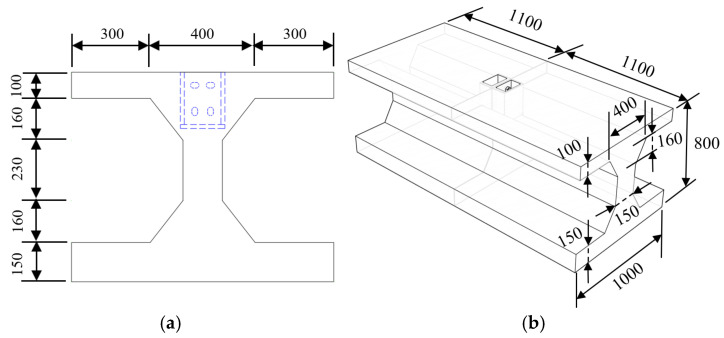
Specimen geometry and structural configuration (unit: mm): (**a**) specimen section diagram and (**b**) stereogram of the test piece.

In order to study the mechanical properties of the shear force of the longitudinal joint, three local specimens of the joint were made. The test parameters were the tightening torques of the high-strength bolt, as shown in Table 1, where *d* is the diameter of the bolt, *T* is the test tightening torque, and *T*_c_ is the standard tightening torque. Considering the conditions of the test site, some structures were intercepted as test bodies according to certain similar conditions on the basis of the prototype structure. The joint force test piece adopted the width of the 1 m range of the longitudinal joint of the inverted arch, while the height direction followed a 1/2 scale of the component. For the opening position of the inverted arch component, the structure was simplified as an I-section. Dimensional analysis determined the similarity coefficients of the local joint specimen and the original structure in geometry, physics, and mechanics. The specific geometric dimensions and structures are shown in Figure 6.

The test piece used C40-grade ordinary concrete. For reinforcement, the transverse members employed 8 mm-diameter HRB400 steel bars, while the abdominal and flange longitudinal reinforcements utilized 10 mm and 14 mm HRB400 steel bars, respectively. The embedded steel box consisted of 15 mm-thick Q355NH weathering steel plates with pre-opened elliptical bolt holes. These holes were arranged with the upper row’s groove holes oriented horizontally and the lower row’s vertically. During fabrication, the formed steel box was welded to the test piece’s corresponding longitudinal reinforcement.

The test employed grade 8.8 M16 large hexagonal high-strength bolts with a pitch of 1.5 mm and effective stress cross-sectional area of 157 mm^2^. The measured yield strength of the bolt was 670.6 MPa, and the ultimate tensile strength was 850.1 MPa. For the weathering steel, the measured yield strength was 458.2 MPa, and the ultimate tensile strength was 562.7 MPa. The steel bar showed a measured yield strength of 432.1 MPa and an ultimate tensile strength of 622.2 MPa. The average compressive strength of the concrete 28-day cube used in the test piece was 39.2 MPa.

**Figure 7:**
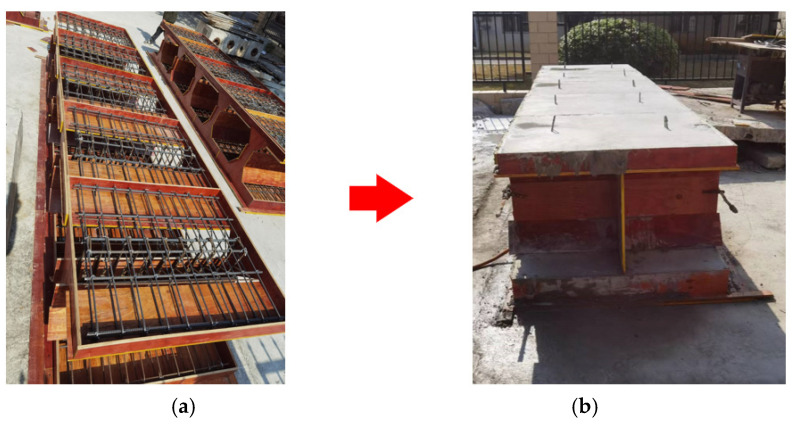
Dimensional detailing of the precast invert arch: (**a**) internal reinforcement cage configuration and (**b**) cast-formed specimen.

The test specimens consisted of two identical components connected by bolts. As shown in Figure 7, specimen fabrication began with template assembly according to a single component’s size and structure, followed by steel cage placement and concrete pouring. After forming, the prefabricated components underwent water curing under natural laboratory conditions. Following 28 days of maintenance, component assembly and combination were performed, with test torque applied to high-strength bolts using a torsion wrench. When the bolts were tightened, a standard tightening torque of about 30% was applied to all bolts, and then the torsion force was applied to the diagonal bolts to the target value.

### 2.4. Test Setup and Loading Scheme

**Figure 8:**
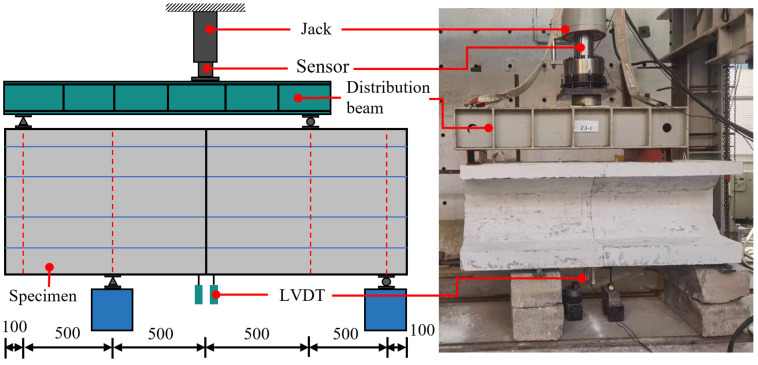
Dimensional detailing of the precast invert arch (unit: mm).

The test was carried out under the 2000 KN electro-hydraulic servo-loading system with a load accuracy of ±1% full scale and a displacement accuracy of ±0.5% full scale. The arrangement of the loading device and displacement meter is shown in Figure 8. During testing, the steel beam was used to control the load of the loading point, so as to achieve the same ratio of the forces of the two loading points and keep the joint in the pure shear state. Two rebound displacement sensors (LVDTs) were installed near the bottom joint of the specimen, with one placed on each side of the joint. The probe tips of the LVDTs were in contact with the underside of the specimen, while their bases were fixed to the ground. Each LVDT had a measuring range of 50 mm and a linear accuracy of ±0.1%. Calibration tests indicated that the measurement error was within ±0.01 mm. No significant drift or hysteresis was observed throughout the loading process.

Prior to formal loading, the specimen underwent preloading at 10% of the anti-slip load specified by the high-strength bolt shear connection theory, completing two full load–unload cycles before main testing commenced. In the formal loading, the load was controlled first, and the loading increment of each stage was 1/20 of the estimated bearing capacity of the specimen. The loading rate was 1 kN/s, and the loading time of each stage was 2 min. After loading to the yield load, the displacement control loading was used, and the control rate was 1 mm/min. The test was terminated when the bearing capacity dropped below 85% of the peak value or the specimen was obviously damaged. The loading rate of the test process was determined by referring to Chen [26] and considering the size and deformation.

## 3. Experimental Results and Analysis

### 3.1. Joint Failure Mode

**Figure 9:**
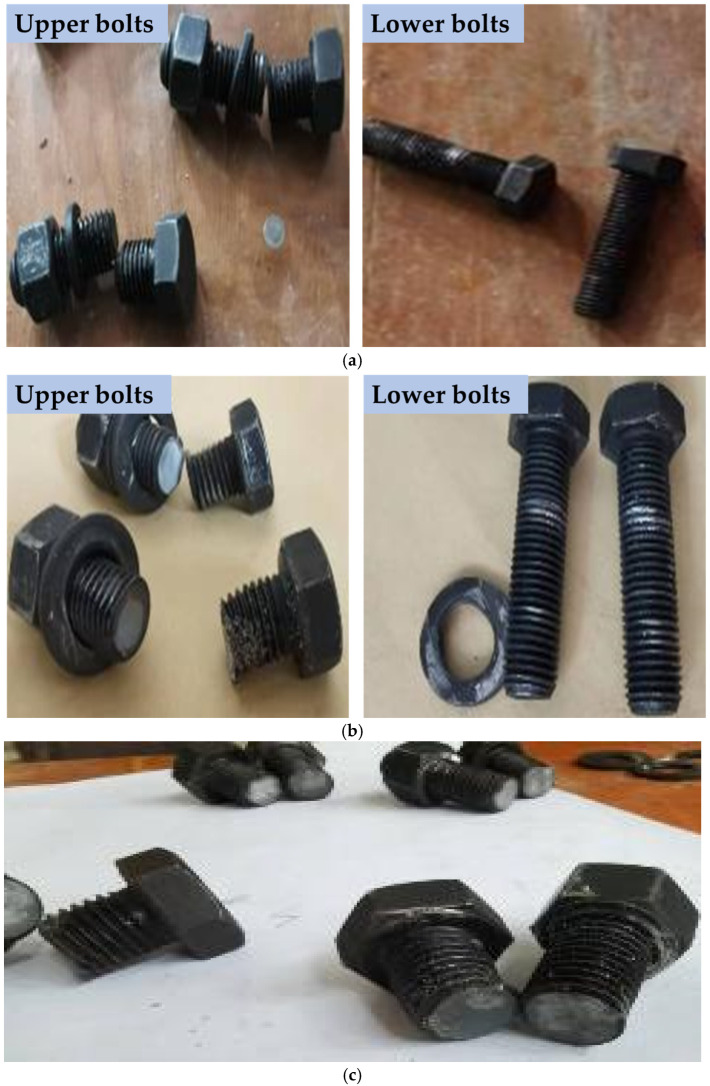
Shear failure of the bolts: (**a**) Z-1, (**b**) Z-2, and (**c**) Z-3.

Experimental observations revealed that the embedded steel boxes and the surrounding concrete at the base of the three sets of longitudinal arch joint specimens remained largely undamaged. In contrast, all three sets exhibited varying degrees of damage or shear failure in the connecting bolts. The failure modes of the high-strength bolts could be broadly categorized into two types:

(1) The two upper bolts were sheared off, while the two lower bolts remained undeformed. For specimen Z-1, the tightening torque was 100 N·m, resulting in a relatively low preloading force. During the initial loading stage, a rapid increase in relative shear displacement was observed at the interface. Upon reaching the ultimate load, the upper two bolts failed by shear, whereas the lower two bolts showed no significant deformation apart from minor thread wear. This behavior can be attributed to the presence of clearance in the bolt holes—specifically, slotted holes approximately 30 mm in length along the loading direction. The detailed failure mode is illustrated in Figure 9a. For specimen Z-2, with a tightening torque of 150 N·m and a moderate preloading force, no significant change occurred during early loading. As the load increased, the failure pattern was again characterized by shear failure of the two upper bolts, while the lower bolts remained intact. The shear fracture surfaces were relatively flat, as shown in Figure 9b.
(2) All four bolts were sheared off. In specimen Z-3, the applied tightening torque was the standard 200 N·m. During the early stage of loading, the relative shear displacement at the interface was small and increased slowly. However, in the later stages, significant shear deformation occurred. At the point of connection failure, all four bolts failed simultaneously in shear. Severe deformation of the washers was observed, and the shear fracture surfaces of the bolts were relatively smooth and flat. The detailed failure mode is illustrated in Figure 9c.

According to Clause 9.2.1.3 of Eurocode 2 [27] and Clause 3.1.2 of the Chinese code GB 50010-2010 Code for Design of Concrete Structures [28], structural joints—especially at critical locations—should exhibit adequate ductile behavior to ensure safety under overload or accidental actions. Although the failure mode observed in specimen Z-3 did not fully meet the current code requirements for ductility, improvements can be achieved by applying mild surface roughening at the interface, using slightly deformable washers, or optimizing the bolt preload. These measures can enhance energy dissipation during the slip process and delay failure, thereby improving overall ductility.

Currently, research on longitudinal joints of assembled tunnel inverts is limited. In this study, the work of Zhang [24] was selected for comparison. Zhang’s study focused on the longitudinal joints within segmental linings of shield tunnels, where long bolt rods were used. The comparison of failure modes is shown in Table 2.

It can be observed that in Zhang’s study [24], the longitudinal joints of tunnel segments exhibited more diverse failure modes compared to those in the present tests. In addition to bolt failure, concrete crushing was also reported. The difference in failure modes may be attributed to the fact that Zhang’s specimens were subjected to both shear and bending, whereas the specimens in this study were subjected to shear only. The discrepancy may also be related to differences in boundary conditions and the use of scaled models in the experimental setup.

### 3.2. Load–Slip Curves

**Figure 10:**
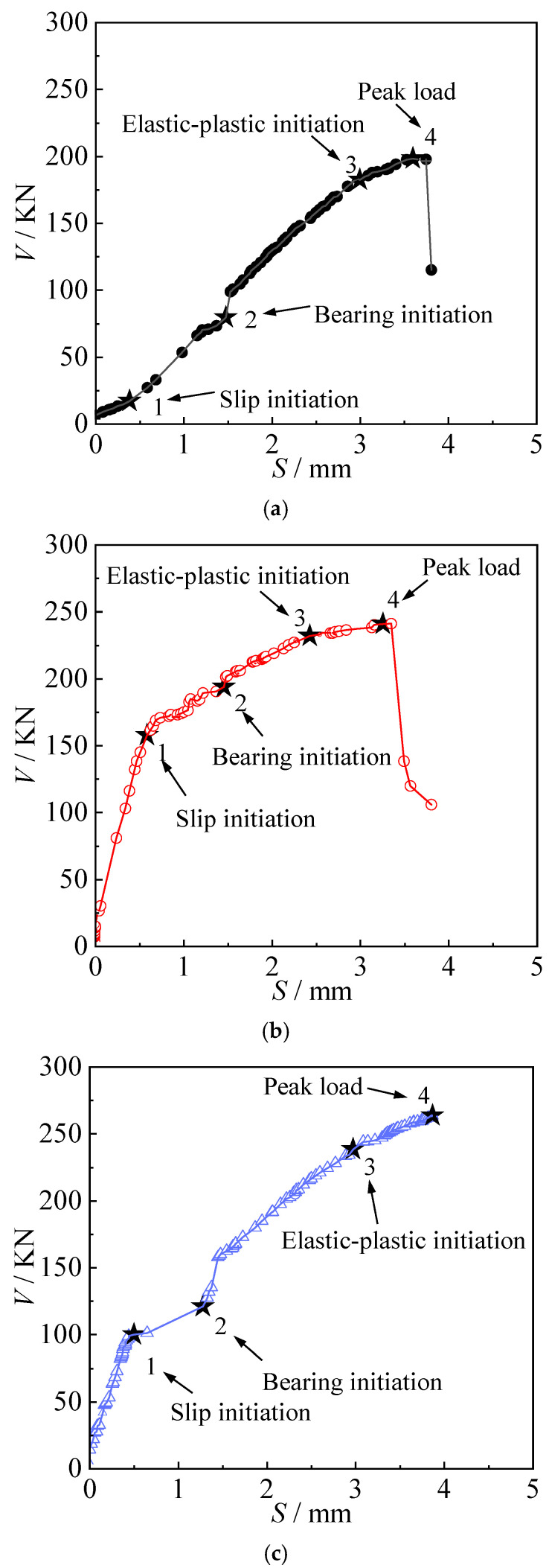
Load–slip curves: (**a**) Z-1, (**b**) Z-2, and (**c**) Z-3.

Figure 10 presents the complete load–slip (*V*-*S*) curves for all longitudinal joints. As shown in the figure, the load–slip response under shear loading exhibited a similar overall trend across all specimens, typically progressing through four distinct stages:

(1) Friction transmission stage: At the initial stage of loading, the applied shear force remained lower than the frictional resistance at the interface between the steel boxes. The gap between the bolt shank and the bolt hole remained unchanged, and the joint behaved elastically. In the *V*-*S* curve, this stage corresponds to the initial linear segment (0–1), indicating a proportional increase in slip with load.
(2) Slip stage: When the applied load exceeded the frictional resistance provided by the high-strength bolt connections, a sudden relative slip occurred at the joint interface until the bolt shank came into contact with the hole wall. This stage is represented by the nearly horizontal segment between points 1 and 2 in the *V*-*S* curve.

In this stage, specimen Z-1 exhibited a slightly different load–slip response compared to specimens Z-2 and Z-3. Its *V*-*S* curve showed an upward sloping linear segment rather than a horizontal plateau. This difference was attributed to the lower bolt-tightening torque applied to Z-1. Even under a relatively small external load, relative sliding at the joint interface began early and continued until the bolt shank contacted the hole wall. During this process, the load was primarily transferred through sliding friction at the arch joint interface. As a result, the transition between the frictional transfer stage and the slip stage was less distinct in specimen Z-1.

Bearing stage: As the load continued to increase, the load–slip curves for all specimens exhibited a noticeable upward inflection when the relative displacement reached approximately 1.5 mm, corresponding to the preset clearance between the upper bolt shank and the hole wall. At this point, the slip increment became minimal, while the load rose rapidly. Due to the elastic properties of the connection materials, the *V*-*S* curves showed a linear ascending trend beyond this inflection, continuing until the elastic limit of the joint was reached (denoted as point “3”). In this stage, the load was primarily transferred through bearing contact between the bolt shank and the hole wall

Elastoplastic stage: In this stage, even a small increase in load resulted in a rapid growth of shear deformation in the connection, ultimately leading to joint failure. The peak point “4” on the load–slip curve represents the ultimate load-bearing capacity of the longitudinal arch joint. After reaching this peak, the bolts failed abruptly in a brittle shear mode, causing a sharp drop in load. Due to the sudden nature of this failure, the descending branches of the curves were difficult to capture accurately for all specimens, as shown in Figure 10.

### 3.3. Characteristic Load Analysis

**Figure 11:**
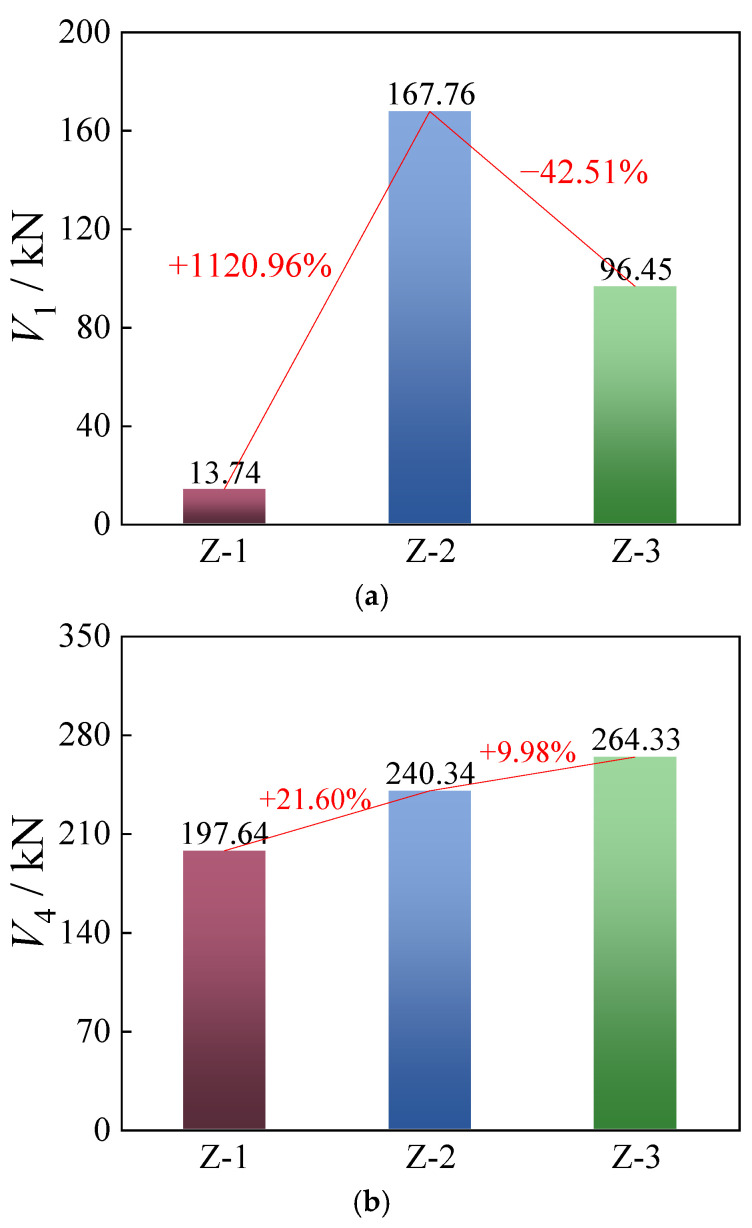
Comparison of ultimate bearing capacity: (**a**) *V*_1_ and (**b**) *V*_4_.

To further investigate the influence of tightening torque on the shear performance of longitudinal arch joints, the frictional limit load and bearing limit load were selected as characteristic indicators of shear capacity. A comparison of the frictional limit load *V*_1_ and the bearing limit load *V*_4_ for each joint is presented in Figure 11.

As shown in Figure 11a, the frictional limit load *V*_1_ of the joints initially increased and then decreased with the tightening torque. Since specimen Z-2 was subjected to a higher torque than Z-1, its initial slip load was approximately 1120.96% greater than that of Z-1. This indicates that a higher tightening torque generally leads to a higher initial slip resistance of the longitudinal joint. However, the initial slip load of specimen Z-3 was approximately 42.51% lower than that of Z-2, despite Z-3 being subjected to an even greater torque. This unexpected reduction may be attributed to fabrication inconsistencies—specifically, a rougher concrete surface at the joint interface in Z-2 compared to Z-3, which could have provided better frictional resistance. This inference is supported by the small-amplitude fluctuations observed in the load–slip curve of Z-2 during the slip stage (between points “1” and “2”), suggesting intermittent micro-slips due to increased surface roughness.

A comparison of the bearing limit loads *V*_4_ revealed that the ultimate load-bearing capacity of specimen Z-2 was approximately 21.60% higher than that of Z-1, while Z-3 exceeded Z-2 by about 9.98% (as shown in Figure 11b). This indicates that applying the standard tightening torque to the bolts of the longitudinal arch joint yielded the highest bearing capacity. The ultimate load-bearing capacity of the joint increased with the tightening torque. This can be attributed to the mechanical behavior of the bolts during the shear-bearing phase: In addition to shear forces, the bolts were subjected to additional bending moments and axial tensile forces induced by shank deformation. A higher tightening torque ensured tighter contact between the connected steel components, which restricted bolt deformation under load. As a result, the additional bending and axial forces experienced by the bolts were reduced, thereby enhancing their shear resistance.

In addition, Figure 11 shows that the differences in ultimate slip displacement among all specimens were relatively small, with none exceeding 1 mm. Nevertheless, it is not recommended to apply excessively high tightening torque to the longitudinal joint bolts. In specimen Z-3, all bolts failed through complete shear fracture, with smooth fracture surfaces, indicating a more pronounced brittle failure tendency compared to specimens Z-1 and Z-2. Based on these findings, it is suggested that in practical engineering applications, a tightening torque of approximately 70% to 85% of the standard torque be applied to longitudinal bolts in arch joints to balance shear performance and failure ductility.

## 4. Calculation of Shear Capacity of Longitudinal Joints in Inverted Arches

Based on experimental observations, the shear behavior of longitudinal arch joints closely resembled that of conventional high-strength bolted connections in steel structures. Therefore, drawing upon existing research on high-strength bolted connections in steel construction, a shear capacity calculation formula is proposed for the prefabricated longitudinal arch joints used in mountain tunnel structures. Since the groundwater in the tunnel is mainly humid and drips, and the surface water body is basically undeveloped, the influence of the groundwater level on the shear-bearing capacity of the anchor rod is not considered. According to design standards, high-strength bolted connections are classified into two types: friction-type and bearing-type connections. In friction-type connections, the design is based on the principle that the applied shear force must not exceed the frictional resistance at the contact surface. In contrast, bearing-type connections permit slip at the interface prior to failure, and the connection is considered to have failed when the bolt shank shears off or the connected plates experience bearing failure

The shear capacity of a single high-strength friction-type bolted connection is given by [29]:

(1) $$N_{m}^{b}=kn_{f}\mu P,$$

where *k* is the hole-type coefficient: 1.0 for standard holes, 0.85 for oversized holes, 0.7 when the force is perpendicular to the long axis of slotted holes, and 0.6 when parallel, *n*_f_ is the number of friction surfaces transmitting the force, with *n*_f_ = 1 for single shear and *n*_f_ = 2 for double shear, *μ* is the slip resistance coefficient of the contact surface, typically taken as 0.3 for untreated surfaces, and *P* is the design value of the preload force applied to a single high-strength bolt.

The relationship between the final tightening torque and the installation preload for large hexagon head high-strength bolts is given by [30]:

(2) $$T_{c}=KP_{c}d,$$

where *T*_c_ is the final tightening torque, *P*_c_ is the installation preload, *K* is the average torque coefficient of the bolt assembly (ranging from 0.11 to 0.15, and taken as 0.15 in this study), and *d* is the nominal diameter of the high-strength bolt. Since preload loss inevitably occurs after tightening, a compensation must be applied to ensure that the assembly reaches the target design preload during service. To account for this, the installation preload was increased by 10% over the design preload, i.e., *P*_c_ = 1.1*P*, and was rounded to the nearest multiple of 5 kN for practical application.

For bearing-type connections using high-strength bolts, the load-bearing behavior after bolt-bearing engagement is similar to that of ordinary bolts. Ordinary bolted connections typically exhibit four potential failure modes: shear failure of the bolt shank, bearing failure at the hole edge, net section fracture of the connected plate, and block shear failure at the plate end. Among these, net section fracture is generally prevented through strength design of the connected members, while block shear failure at the plate end can be avoided by adhering to the detailing requirements for fastener connections specified in relevant design codes. Therefore, in the shear capacity calculation of high-strength bearing-type bolted connections, only shear failure of the bolt shank and bearing failure at the hole edge are considered.

In tunnel prefabricated inverted arch longitudinal bolted connections, the steel boxes are pre-embedded in concrete. If structural damage occurs during assembly, the cost of repairing or replacing the embedded steel boxes would be significantly higher than that of replacing bolts. Therefore, bearing failure of the hole edge in the steel box panels must be avoided, and only shear failure of the bolt shank is permitted at the longitudinal joint. Based on this design requirement, the shear capacity of a single high-strength bolt in a bearing-type connection within the inverted arch longitudinal joint is calculated as follows [31]:

(3) $$N_{V}^{b}=\frac{1}{\sqrt{3}}n_{v}\frac{\pi d^{2}}{4}f_{y}^{b},$$

where *n*_v_ is the number of shear planes, *n*_v_ = 1 for single shear, *n*_v_ = 2 for double shear, and *n*_v_ = 4 for quadruple shear, and *d* is the nominal diameter of the high-strength bolt. If the shear plane passes through the threaded portion, *d* should be taken as the effective diameter at the threads. *f*_y_ is the yield strength of the bolt material.

Based on the observed failure modes of the connections, when the applied tightening torques were 100 N·m and 150 N·m, the two upper bolts in the connection were sheared off, while the lower bolts only experienced positional slip without shear failure. Therefore, the shear capacity of the longitudinal joint should be considered as the sum of the bearing capacity of the sheared bolts and the frictional resistance of the bolts that remained intact:

(4) $$N=mN_{m}^{b}+nN_{v}^{b}=m\frac{kn_{f}\mu T_{c}}{1.1Kd}+n\frac{n_{v}\pi d^{2}f_{y}^{b}}{4\sqrt{3}},$$

where *m* is the number of bolts with internal force acting perpendicular to the slot direction and *n* is the number of bolts with internal force acting parallel to the slot direction.

A comparison between the calculated shear capacities of the joints, obtained using Equation (4), and the experimental results is presented in Table 3. In the table, $N_{u}^{e}$ is the experimental value, and $N_{u}^{c}$ is the calculated value. As shown in the table, all calculated values were lower than the corresponding experimental values, with the ratio of calculated to experimental results ranging from 0.69 to 0.86. The average ratio of the theoretical shear capacity to the experimental results was approximately 0.76, indicating that the current theoretical model provided a conservative estimate to a certain extent. This conservative bias may be attributed to several factors: (1) The simplified calculation neglected the combined contribution of friction and bearing action at the bolt–concrete interface. (2) The estimated efficiency of preload transfer was relatively low, without fully accounting for the influence of local contact stiffness. (3) The additional shear resistance resulting from confinement effects near the bolt region was not considered. Moreover, the model assumed an ideal uniform stress distribution, which did not reflect the stress concentrations observed around the bolt holes during testing.

**Figure 12:**
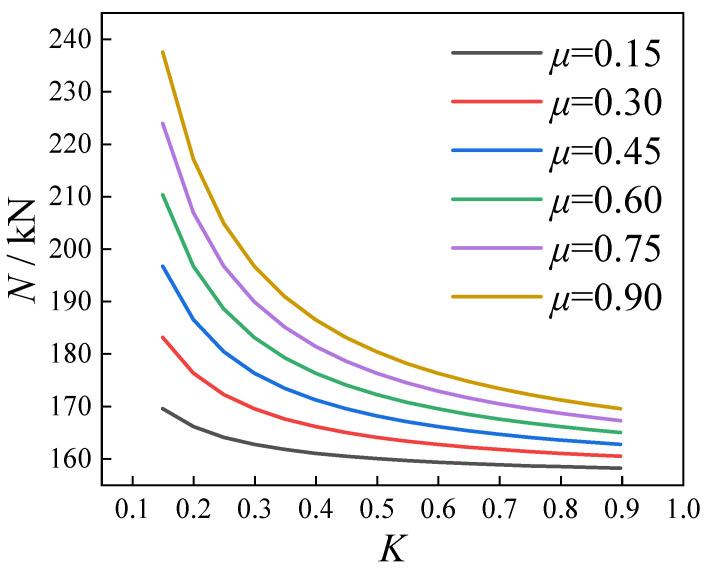
The influence of different friction coefficients and torque coefficients on the calculation results.

The influence of different friction coefficients (*μ*) and torque coefficients (*K*) on the calculation results was investigated, as illustrated in Figure 12. It can be observed that when the torque coefficient was low, variations in the friction coefficient led to significant changes in the calculated results. In contrast, when the torque coefficient was high, the impact of the friction coefficient on the calculation results became progressively smaller.

## 5. Conclusions

Based on shear tests conducted on three longitudinal joint specimens, this study investigated the influence of high-strength bolt-tightening torque on the failure mode, full-range load–slip behavior, and shear capacity of the longitudinal joints. Finally, a shear capacity calculation formula for the tunnel invert longitudinal joint was proposed. Based on the results of this study, the following conclusions can be drawn:

(1) For all three groups of local longitudinal joint specimens subjected to shear failure, no significant damage was observed in components other than the high-strength bolts. In specimens where 50% and 70% of the standard tightening torque was applied, the upper two bolts were sheared off, while the lower two only showed thread damage, indicating that the joint did not fail completely at once. In contrast, for the specimen with bolts tightened to the full standard torque, all bolts were sheared off, leading to a complete and sudden failure of the joint.
(2) The shear capacity of the longitudinal joint increased with the tightening torque applied to the high-strength bolts. However, joints assembled with bolts tightened to the full standard torque exhibited brittle failure. Therefore, it is recommended that a tightening torque of approximately 70% to 85% of the standard value be used in practical engineering applications. It is noted that the lower limit of the tightening torque range is based on the principle of ensuring that both rows of bolts can give full play to their strength. The value should be greater than 70%, and it was taken as 70% in this paper. To maintain a safety margin and prevent simultaneous shear failure in both bolt rows, the upper threshold was conservatively selected at the midpoint of the range of 70% to 100%, corresponding to 85% of the maximum capacity. Due to the small number of test specimens, the lower limit value of 70% may be more conservative. More tests will be added to further determine the value more accurately. It is further recommended to include some safety factors in the design to ensure that there is sufficient reserve for shear slip or failure of the joint. The upper limit is the same.
(3) Based on the observed failure modes of the longitudinal joints and existing research on bolted connections in steel structures, a shear capacity calculation formula for the tunnel invert longitudinal joint was proposed. The calculated results were conservative, indicating a safe-side estimation.

It should be noted that there are some limitations in this experiment. There were some differences between the 1/2 scale model and the actual structure. The stress distribution was not necessarily scaled, especially in stress concentration areas, such as bolt holes. The size effect may lead to local stress response amplification. At the material level, three specimens were tested in this paper. The limited sample size, coupled with the single material properties of bolts and concrete, requires caution when inferencing results. There was less variable analysis of the test, and the reference to the influence of different strength materials on the specimens was limited. Subsequent studies carried out finite element simulation based on the results of this test to correct the size effect and provide support for the establishment of size-independent design formulas. At the same time, different strength materials will be introduced for experimental comparison and expansion analysis, in order to provide practical engineering reference in terms of economic benefits and construction efficiency.

## Figures and Tables

**Table 1:** Parameters of specimens.

Specimen	*d* (mm)	*T* (N·m)	*T*_c_ (N·m)	*T*/*T*_c_
Z-1	16	100	200	50%
Z-2	16	150		75%
Z-3	16	200		100%

**Table 2:** The comparison of failure modes.

Study	Joint Type	Specimen	Bolt Specification	Failure Mode
This study	Longitudinal joint of prefabricated assembled tunnel inverts	Z-1	M16	Shearing of upper-row bolts
		Z-2	M16	Shearing of upper-row bolts
		Z-3	M16	All bolts sheared off
Zhang [24]	Longitudinal joint of shield tunnel segments	PBM-1	M42	Concrete crushing failure
		PBM-2	M42	Cracking at the end of the specimen
		PBM-3	M42	Thread stripping or bolt fracture failure
		PBM-4	M42	Thread stripping or bolt fracture failure
		PBM-5	M42	Cracking on the inner surface
		PBM-6	M42	Cracking on the inner surface and at the joint interface

**Table 3:** Comparison between calculated and experimental shear capacities of the joints.

Specimens	$N_{u}^{e}$/KN	$N_{u}^{c}$/KN	$N_{u}^{c}$ $/N_{u}^{e}$
Z-1	197.64	169.33	0.86
Z-2	240.34	176.15	0.73
Z-3	264.33	182.96	0.69
Average			0.76

## Data Availability

The original contributions presented in this study are included in the article. Further inquiries can be directed to the corresponding author.
